# Data on full stationary wave-field measurement of a suspended steel plate punctually loaded

**DOI:** 10.1016/j.dib.2024.111180

**Published:** 2024-12-09

**Authors:** Xuefeng Li, Thomas Brion, Pascal Fossat, Mohamed Ichchou, Olivier Bareille, Abdel-Malek Zine

**Affiliations:** a LTDS, UMR-CNRS 5513, École Centrale de Lyon, 36 Avenue Guy de Collongue, 69134, Écully, France; b Institut Camille Jordan, UMR-CNRS 5208, École Centrale de Lyon, 36 Avenue Guy de Collongue, 69134, Écully, France; c The Marcus Wallenberg Laboratory for Sound and Vibration Research (MWL), Department of Engineering Mechanics, KTH Royal Institute of Technology, Stockholm, Sweden

**Keywords:** Vibroacoustics, Frequency response, Waveform, Full vibration field

## Abstract

The dataset presented contains the experimental structural response, in the frequency domain, of a suspended steel plate to a point force excitation. The plate is excited by a mechanical point force generated by a Brüel & kJær shaker with a white noise signal input from 3.125 Hz to 2000 Hz. The out-of-plate displacement fields on a 2D grid were measured using a Polytec PSV-400 Scanning Vibrometer. Finally, the displacement fields are acquired by a Fourier analyser connected to a sampler. The dataset provided is a useful resource for researchers to study the structural dynamic behaviour of large thin plates in the frequency domain and to validate the effectiveness of wavenumber identification methods. Its value has been illustrated in the research paper “Algebraic K-Space Identification 2D technique for the automatic extraction of complex k-space of 2D structures in presence of uncertainty” [1]. The data collection was carried out during three weeks in April 2022 at the Ecole Central de Lyon.

Specifications Table

**Table:** 

Subject	Mechanical Engineering
Specific subject area	Vibroacoustics, Wave propagation, Full vibration field
Type of data	Figures, 1D and 3D double array .mat files
Data collection	An electrodynamic shaker (Brüel & kJær, 4810) was used to generate a point mechanical force. The shaker was attached to the structure using an impedance head and driven by a white noise signal over the frequency range from 3.125 Hz to 2000 Hz. A Polytec Scanning Vibrometer (PSV-400) was used to measure the full vibration field. Displacement fields are acquired by a Fourier analyser installed in the measuring instruments.
Data source location	LTDS, UMR-CNRS 5513, École Centrale de Lyon, Écully, France.
Data accessibility	Data identification number: 10.17632/snd9j7y5hn.3 Direct URL to data: 10.17632/snd9j7y5hn.3
Related research article	Brion T, Li X, Fossat P, Ichchou M, Bareille O, Zine A-M. Algebraic K-Space Identification 2D technique for the automatic extraction of complex k-space of 2D structures in presence of uncertainty. Mechanical Systems and Signal Processing

## 1 Value of the Data

• The dataset provided is a valuable resource for researchers studying the structural dynamic behaviour of the large thin plates in the frequency domain. In particular, it can be used to improve a study by adding an experimental validation or comparison.
• The researchers can use the provided dataset as input to extract wavenumbers or wave dispersion characteristics in general and experimentally validate the accuracy and robustness of their proposed inverse method.
• The load-displacement is a raw data, so the dataset can be reprocessed or reused directly for different purposes.

## 2 Background

The identification of the wave and energy propagation of 2D structures is of great importance for the analysis of their dynamic behaviour [2,3]. The main purpose of this experiment was to measure under real conditions the efficiency and robustness of a new 2D inverse vibroacoustic method for estimating wave propagation properties from a two-dimensional wave field [1]. Previously, this method had only been tested with analytical and numerical test cases. There have been many inverse methods for wave propagation characterisation, which can be categorised as follows:

1) The Fourier-based methods: the Fourier transform itself [4], the McDaniel's method [5], the Inhomogeneous Wave Correlation (IWC) method [6] or the Spatial LAplace Transform for COmplex Wavenumber (SLaTCoW) method [7].
2) The Prony-based methods: the High Resolution Wavevector Analysis (HRWA) [8], the INverse COnvolution MEthod (INCOME) [9] or the Bloch wavenumber identification method [10].
3) Other principle-based methods: the image sources method [11], the Force Analysis Technique (FAT) [12] or the maximum likelihood estimation [13].

## 3 Data Description

The displacement fields, measured from the excited steel plate, are presented for 800 frequencies from 3.125 Hz to 2500 Hz with a step of 3.125 Hz. At each frequency, the displacement field is known on a grid of 53×55 points sampling an area of a 0.825 m by 0.535 m.

The data are in the Matlab file “Data_steel_plate.mat” which contains:

• “Frequencies” (800 double array) are the frequencies sampled from 3.125 Hz to 2500 Hz with a frequency step of 3.125 Hz.
• “X” (53 double array) are the positions of the data points in the x-direction.
• “Y” (55 double array) are the positions of the data points in the y-direction.
• “Displacement_fields” (53×55×800 double array) are the displacement value at each abscissa, ordinate and frequency.

The Matlab script “Plot_displacement_field.m” is also provided and allows the displacement field at different frequencies to be plotted from the data.

**Fig. 1:**
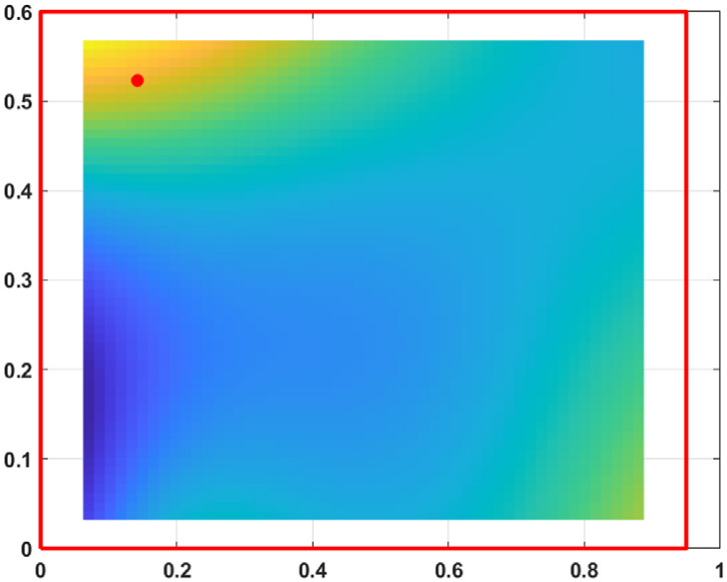
Displacement field at 25 Hz.

**Fig. 2:**
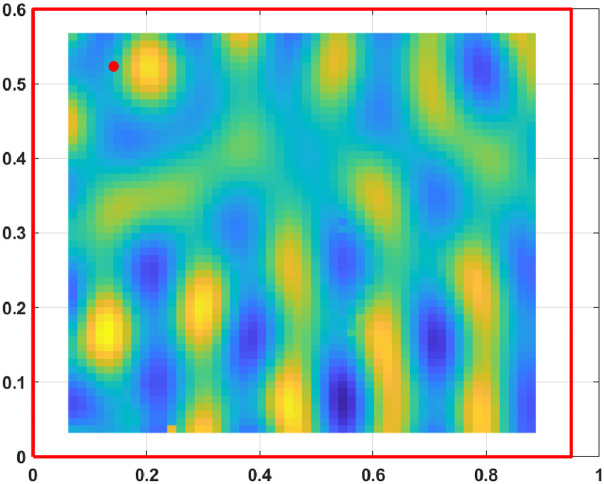
Displacement field at 750Hz.

**Fig. 3:**
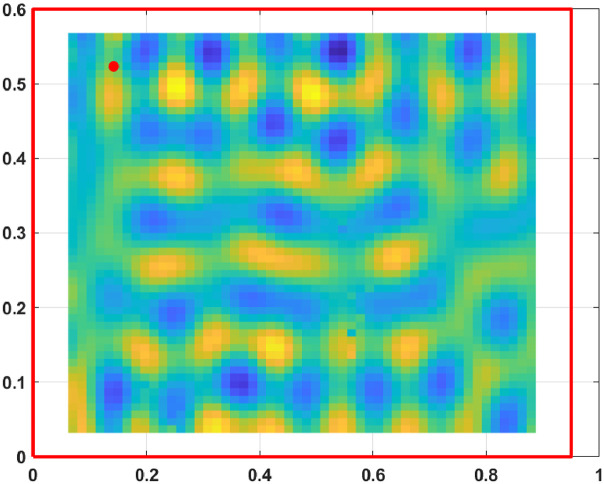
Displacement field at 1500 Hz.

**Fig. 4:**
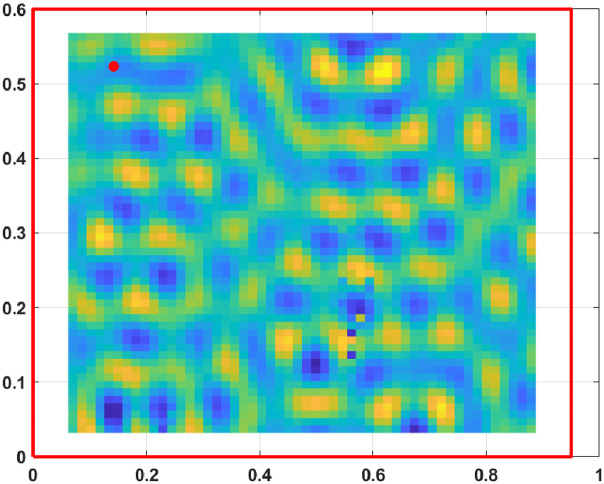
Displacement field at 2500 Hz.

As an example, the displacement fields at 25 Hz, 750 Hz, 1500 Hz and 2500 Hz are provided Fig. 1, Fig. 2, Fig. 3, Fig. 4. The red dot is the position of the excitation point and the red lines are the plate boundaries.

## 4 Experimental Design, Materials and Methods

The plate is a 0.95 m by 0.6 m steel plate with a thickness of 2mm. The plate is suspended from a fixed frame using polylactic acid filaments to simulate free-free boundary conditions.

An electrodynamic shaker (Brüel \& kJær, 4810) is used to generate a point mechanical force at the red point shown in Fig. The shaker is attached to the structure using an impedance head and is driven by a white noise signal over the frequency range from 0 Hz to 2500 Hz.

**Fig. 5:**
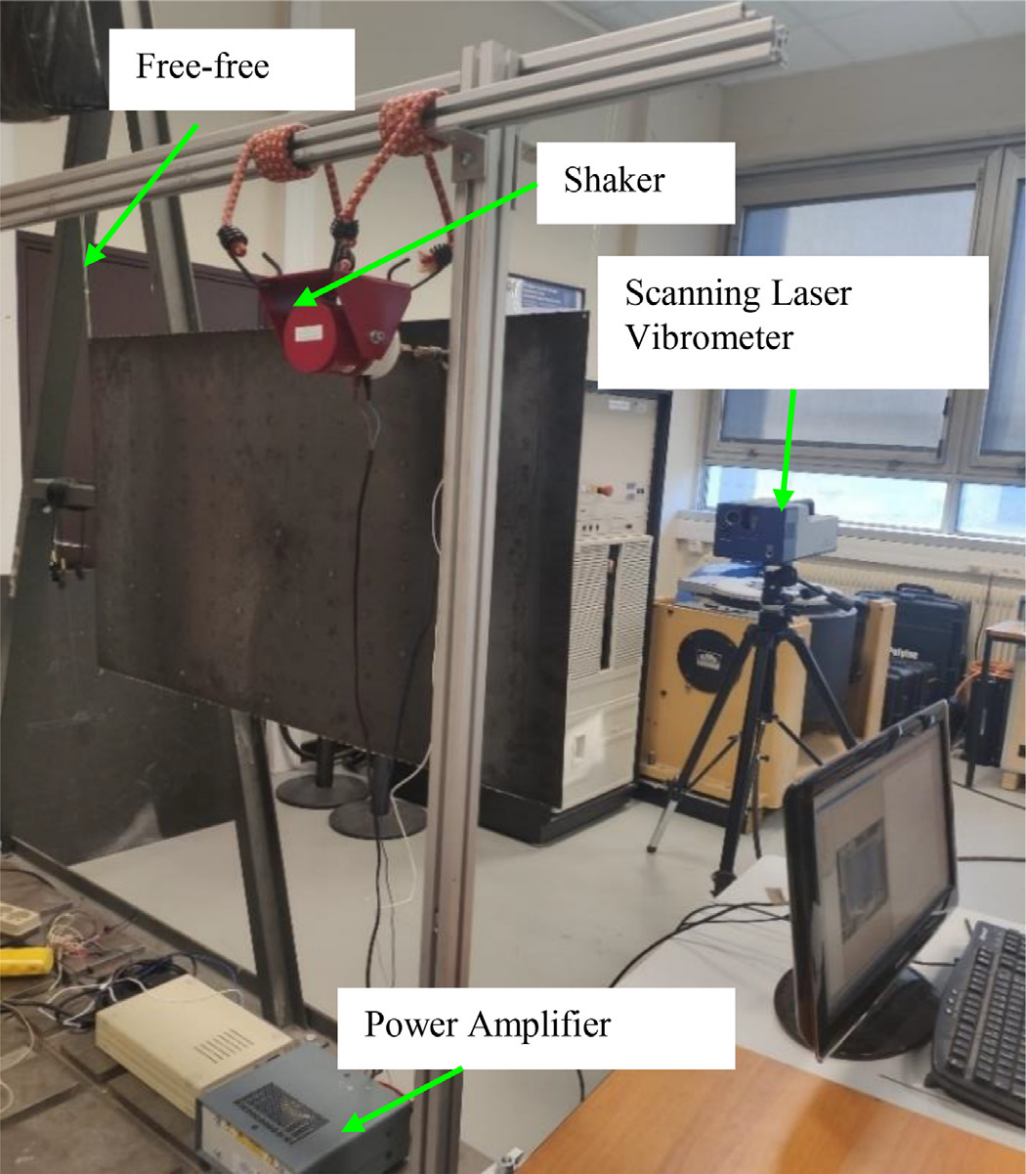
Picture of the plate and the experimental setup [1].

A Polytec Scanning Vibrometer (PSV-400) was used to measure the full vibration field on a 53×55 mesh (sampling interval Δx=1.59 cm and Δy=0.99 cm). The scanning area of 0.825 m by 0.535 m is shown in the blue dotted rectangular box of Fig. 5. The vibrometer (PSV-400), was placed approximately 1.2 m from the plate.

Finally, the displacement fields are acquired by a Fourier analyser installed in the measuring equipment.

The vibrometer has the following setting parameters:

• Windowing: Hanning window,
• Overlap percentage: 75 %,
• Number of averages: 30,
• Method for averaging: Exponential averaging,
• Filtering: Band-pass filter.

The Hanning window was selected because it can reduce the spectral leakage effects and improve the frequency resolution.

The overlap percentage of 75 % was chosen to ensure that each segment of data in the time domain is adequately represented in the frequency domain, thereby improving the accuracy of the spectral estimation.

A number of averages of 30 was selected to compromise between the improvement of the signal-to-noise ratio and data acquisition time. A number of 30 provides an acceptable displacement field.

The Exponential averaging gives more weight to recent measurements, making it well suited for dynamic systems, and also maintains a reasonable smoothing effect on the data.

The Band-pass filter allows to isolate the frequency range of interest, removing low and high-frequency noise effectively.

## Limitations

None.

## Ethics Statement

The authors confirm that they have read and follow the ethical requirements for publication in Data in Brief and confirm that the current work does not involve human subjects, animal experiments, or any data collected from social media platforms.

## CRediT authorship contribution statement

**Xuefeng Li:** Investigation, Resources, Data curation, Writing – review & editing. **Thomas Brion:** Software, Writing – original draft, Writing – review & editing. **Pascal Fossat:** Writing – review & editing. **Mohamed Ichchou:** Conceptualization, Writing – review & editing, Validation, Project administration, Funding acquisition. **Olivier Bareille:** Writing – review & editing, Supervision, Project administration, Funding acquisition. **Abdel-Malek Zine:** Writing – review & editing, Supervision, Project administration, Funding acquisition.

## Data Availability

Mendeley DataData on full stationary wave-field measurement of a suspended steel plate punctually loaded (Original data).
